# Development of Chloroplast Microsatellite Markers and Analysis of Chloroplast Diversity in Chinese Jujube (*Ziziphus jujuba* Mill.) and Wild Jujube (*Ziziphus acidojujuba* Mill.)

**DOI:** 10.1371/journal.pone.0134519

**Published:** 2015-09-25

**Authors:** Jian Huang, Xiaoting Yang, Chunmei Zhang, Xiao Yin, Shipeng Liu, Xingang Li

**Affiliations:** 1 Research Center for Jujube Engineering and Technology of State Forestry Administration, College of Forestry, Northwest A&F University, Yangling, China; 2 Key Laboratory of jujube for Shaanxi Province, College of Life Science, Yan’an University, Yan’an, China; 3 Key Comprehensive Laboratory of Forest for Shaanxi Province, College of Forestry, Northwest A&F University, Yangling, China; Aristotle University of Thessaloniki, GREECE

## Abstract

*Ziziphus* is an important genus within the family Rhamnaceae. This genus includes several important fruit tree species that are widely planted in China and India, such as the Chinese jujube (*Ziziphus jujuba* Mill.), the wild jujube (*Z*. *acidojujuba*), and the Indian jujube (*Z*. *mauritiana*). However, information about their domestication based on the chlorotype diversity of Chinese jujube population is lacking. In this study, chloroplast microsatellite (cpSSR) markers were developed and used to investigate the genetic relationships between and domestication of jujube cultivars and wild jujube populations. Primer sets flanking each of the 46 cpSSR loci in non-coding regions of the chloroplast genome sequence of *Z*. *jujuba* Mill. cv. ‘Junzao’ were designed. In total, 10 markers showed polymorphisms from 15 samples (9 jujube cultivars and 6 wild jujube individuals), of which 8 loci were due to variations in the number of mononucleotide (A/T) repeats and 2 were due to indels. Six cpSSR markers were used in further analyses of 81 additional samples (63 jujube cultivars, 17 wild jujube samples, and 1 Indian jujube). Using these cpSSR markers, the number of alleles per locus ranged from two to four. In general, the Shannon Index (*I*) for each cpSSR ranged from 0.159 to 0.1747, and the diversity indices (*h*) and *uh* were 0.061 to 0.435 and 0.062 to 0.439, respectively. Seven chlorotypes were found; the Indian jujube showed distinct chlorotypes, and both the Chinese and wild jujube had four chlorotypes and shared two chlorotypes. A dominant chlorotype (G) accounted for 53 of 72 jujube cultivars and 13 of 23 wild jujube individuals. All chlorotypes were highly localized along the Yellow River, from the mid- to the lower reaches, suggesting a wide origin of jujube. These cpSSR markers can be applied to population and evolution studies of Chinese jujube and wild jujube.

## Introduction

*Ziziphus* is an important genus in the family Rhamnaceae [1]. Three economically important species in this genus are widely cultivated: the Chinese jujube (*Z*. *jujuba*), the wild (or sour) jujube (*Z*. *acidojujuba*), and the Indian jujube (*Z*. *mauritiana*). Chinese jujube (hereafter referred to as jujube) is believed to be native to China and is often considered as the generic species of the genus *Ziziphus* [2]. Jujubes are among the most popular native fruit trees in China and have been cultivated for dietary and medical uses for more than 3000 years; they are widely embedded in traditional Chinese culture [3]. At present, jujube plantations cover more than 1.53 million *ha* according to the China Forestry Yearbook 2013, and jujube is the fourth highest-ranking fruit produced in terms of economic importance after the apple, pear, and grape in the temperate regions of China. Wild jujube has a close morphological resemblance to jujube and is often regarded as the wild ancestor of jujube; its seeds have high medicinal value, and it is widely used as the rootstock for jujube [3]. Jujube and wild jujube have been treated as two independent species [1]; however, the taxonomic delineation between them is still debated, and the history of the domestication of jujube remains unresolved.

Many molecular tools have been utilized to study the phylogenetic and population structures of and genetic relationships between jujube and wild jujube. Islam and Simmons (2006) performed an intrageneric classification of 19 species by simultaneous analysis of the morphological characteristics and molecular methods based on nuclear rDNA internal transcribed spacers, 26S rDNA, and the plastid *trnL-F* intergenic spacer; *Z*. *jujuba* and *Z*. *acidojujuba* were tightly clustered into one group [2]. Since 2000, a great deal of research has focused on the genetic relationships between different jujube cultivars and/ or wild jujube individuals using molecular markers, including random amplified polymorphic DNA (RAPD), amplified fragment length polymorphisms (AFLP), sequence-related amplified polymorphisms (SRAP), and simple sequence repeats (SSR) [4–9]. However, despite being excellent markers for use in most analyses, SSR, RAPD, AFLP, and SRAP markers are derived from the nuclear genome and are not suitable for phylogenetic analyses between different species or genera because of their high rate of sequence evolution, which prevents comparisons between sequences and allele sizes above the species level [10, 11]. In contrast, chloroplast SSRs (cpSSR) derived from the chloroplast genome represent ideal complementary molecular tools as nuclear genetic markers. This is because the SSR loci in the chloroplast genome are often distributed throughout the non-coding regions and show higher sequence variations than do the coding regions on the background of a low evolutionary rate and an almost nonexistent recombination rate in chloroplast DNA [11–14]. Therefore, cpSSR markers can be used to investigate population genetics and biogeography and unravel the genetic relationships of closely related species.

In combination with nuclear SSR markers, cpSSR markers have a high discrimination capability for investigating the domestication history, sites of origin, and genetic relationships of cultivated fruit trees, such as grapes [15], citrus [16,17], almonds [18,19], and chestnuts [20]. It is therefore necessary to develop cpSSR markers for jujube to investigate the domestication processes of and genetic relationships between different jujube cultivars and wild jujube individuals.

Historically, because chloroplast genome sequences were unavailable, the development of cpSSR markers relied on universal primer sets that had previously been successfully utilized to amplify cpSSR markers in other species. This strategy proved to be simple and low-cost; however, the primers were not always successful for DNA amplification or for use in the detection of further polymorphisms, making a global analysis of the SSR loci in the chloroplast genome impossible [21]. More recently, the rapid increase in sequencing technologies has led to the discovery of additional plant chloroplast genome sequences, improving the efficiency of developing cpSSR markers by making it possible to directly search for the SSR loci in the chloroplast genome [22–24]. In this study, the SSR loci were identified based on a draft chloroplast genome sequence of the jujube cultivar “Junzao,” which was assembled from sequence reads produced by an Illumina HiSeq 2000 platform, by sequencing the total DNA of young leaves. The polymorphisms in those SSR loci were evaluated using several jujube cultivars and wild jujube individuals. Furthermore, using the SSR markers developed, the chlorotypes of 72 jujube cultivars and 23 wild jujube individuals that originated from China on a national scale were analyzed. The aim of this study was to identify several polymorphic cpSSR markers for use in the analysis of the genetic relationships and domestication patterns between jujube and wild jujube plants.

## Materials and Methods

### Plant materials and DNA extraction

In total, 96 samples were collected: 72 cultivars from jujube (*Z*. *jujuba*), 23 from wild jujube (*Z*. *acidojujuba*), and 1 individual of *Z*. *mauritiana* Lam (Table 1). Young leaves were collected from May to October 2012 from a germplasm collection garden and from a local site. The plant material was acquired with permissions from the National Crop (Jujube) Germplasm Resources Infrastructure (Taigu, Shanxi), National Key Base for Improved Chinese Jujube Cultivar (Cangzhou, Hebei), Jujube Germplasm Repository of Shandong institute of pomology (Taian, Shandong), Jujube experimental station of Northwest A&F University (Qingjian, Shaanxi) and those from private owners abiding by the laws in China. All plant materials used in this study did not involve endangered or protected species.

**Table 1:** Jujube cultivars and wild jujube individuals used in the characterization of the cpSSR markers.

No.	Common names	Taxa	Sampling sites	Original sites	Coordinates of original location	Chlorotypes
1	Maoyezao	*Z*. *mauritiana*	Jinghong,Yunnan	India	N 22.02, E 100.78	A
2	Guizhouxiaozao	*Z*. *jujuba*	Zhengfeng, Guizhou	Zhengfeng, Guizhou	N 25.39, E 105.78	G
3	Zunyitianzao	*Z*. *jujuba*	NKIJ (C371)	Zunyi, Guizhou	N 27.68, E 106.81	G
4	Lianxianmuzao	*Z*. *jujuba*	NCGRI (ZF0173)	Lianxian, Guangdong	N 24.83, E 112.57	E
5	Guanyangchangzao	*Z*. *jujuba*	NCGRI (ZF0145)	Guanyang, Guangxi	N 25.61, E 111.13	G
6	Guanyangduanzao	*Z*. *jujuba*	NCGRI (ZF0146)	Guanyang, Guangxi	N 25.46, E 110.83	G
7	Daguosuanpanzao	*Z*. *jujuba*	Xupu, Hunan	Xupu, Hunan	N 27.88, E 110.57	G
8	Xiaoguosuanpanzao	*Z*. *jujuba*	Xupu, Hunan	Xupu, Hunan	N 27.82, E 110.63	G
9	Hunanniunaizao	*Z*. *jujuba*	Xupu, Hunan	Xupu, Hunan	N 27.85, E 109.8	G
10	Guanyinzao	*Z*. *jujuba*	NCGRI (ZF0147)	Xupu, Hunan	N 28.03, E 110.7	G
11*	Hunanjidanzao	*Z*. *jujuba*	Qidong, Hunan	Qidong, Hunan	N 26.93, E 111.7	G
12	Yiwudazao	*Z*. *jujuba*	NKIJ (C019)	Yiwu, Zhejiang	N 29.4, E 120.1	E
13	Shengxianbaipu	*Z*. *jujuba*	NKIJ (C060)	Chengxian, Zhejiang	N 29.63, E 120.91	E
14	Nanjingzao	*Z*. *jujuba*	NKIJ (C093)	Lanxi, Zhejiang	N 29.32, E 119.6	G
15	Beibeixiaozao	*Z*. *jujuba*	NCGRI (ZF0124)	Beibei, Chongqing	N 29.84, E 106.49	G
16	Fengjiejidanzao	*Z*. *jujuba*	JESN	Fengjie, Chongqing	N 29.57, E 106.85	G
17	Suixiandazao	*Z*. *jujuba*	JGSD	Suixian, Hubei	N 31.84, E 113.28	G
18	Sunanbaipuzao	*Z*. *jujuba*	NKIJ	Suzhou, Jiangsu	N 31.62, E 120	G
19	Jianghuai No.1	*Z*. *jujuba*	JESN	Jiangsu	N 33.37, E 118.05	G
20	Qiyuesu	*Z*. *jujuba*	JESN	Jiangsu	N 33.4, E 118.08	G
21	Xuanchengyuanzao	*Z*. *jujuba*	JGSD	Xuancheng, Anhui	N 30.75, E 118.83	G
22	Fuyangmayizao	*Z*. *jujuba*	NKIJ	Fuyang, Anhui	N 33.02, E 115.73	G
23	Jinximuzao	*Z*. *jujuba*	NCGRI (ZF0352)	Jinxi, Liaoning	N 41.85, E 120.72	G
24	Jinlingchangzao	*Z*. *jujuba*	NCGRI (ZF0351)	Chaoyang, Liaoning	N 41.63, E 120.24	G
25	Jinlingyuanzao	*Z*. *jujuba*	NCGRI (ZF0398)	Chaoyang, Liaoning	N 41.83, E 120.34	G
26*	Xiaopingding	*Z*. *jujuba*	NCGRI (ZF0397)	Chaoyang, Liaoning	N 40.98, E 119.24	G
27	Langjiayuanzao	*Z*. *jujuba*	NKIJ (C131)	Chaoyang, Beijing	N 40.24, E 116.22	G
28	Gagazao	*Z*. *jujuba*	NKIJ	Beijing	N 40.24, E 116.22	G
29	Tianjingusuanzao	*Z*. *acidojujuba*	Tianjing	Tianjing	N 38.99, E 117.5	G
30	Beijinggusuanzao	*Z*. *acidojujuba*	Beijing	Beijing	N 39.9, E 116.42	D
31	Chengtuozao	*Z*. *jujuba*	NKIJ (C049)	Cangzhou, Hebei	N 38.37, E 116.55	G
32	Xingtaigusuanzao	*Z*. *acidojujuba*	Xingtai, Hebei	Xingtai, Hebei	N 37.22, E 114.29	D
33	Xingtaigongsuanzao	*Z*. *acidojujuba*	Xingtai, Hebei	Xingtai, Hebei	N 37.22, E 114.29	G
34*	Dongzao	*Z*. *jujuba*	JGSD	Zhanhua, Shandong	N 37.69, E 118.12	E
35*	Jinsixiaozao	*Z*. *jujuba*	NKIJ (C050)	Shandong/Hebei	N 37.82, E 117.19	G
36*	Pozao	*Z*. *jujuba*	NKIJ (C247)	Shandong/Hebei	N 38.5, E 114.62	D
37*	Zanhuangsuanzao No.1	*Z*. *acidojujuba*	Zanhuang, Hebei	Zanhuang, Hebei	N 37.53, E 114.26	C
38	Zanhuangdazao	*Z*. *jujuba*	Zanhuang, Hebei	Zanhuang, Hebei	N 37.75, E 114.34	G
39	Zanhuangsuanzao No.2	*Z*. *acidojujuba*	Zanhuang, Hebei	Zanhuang, Hebei	N 37.52, E 114.29	G
40	Zanhuanggusuanzao	*Z*. *acidojujuba*	Zanhuang, Hebei	Zanhuang, Hebei	N 37.53, E 114.25	C
41	Malianxiaozao	*Z*. *jujuba*	NKIJ	Zaoqiang, Hebei	N 38.36, E 116.69	D
42	Mopanzao	*Z*. *jujuba*	JGSD	Shandong/Shaanxi/Gansu/Hebei	N 35.72, E 107.72	G
43	Shandonglizao	*Z*. *jujuba*	JGSD	Shandong/Hebei	N 37.82, E 117.62	G
44	Changhongzao	*Z*. *jujuba*	Ningyang, Shandong	Ningyang, Shandong	N 35.77, E 116.96	E
45	Yuanlingzao	*Z*. *jujuba*	Ningyang, Shandong	Ningyang, Shandong	N 36.61, E 116.17	G
46	Huizao	*Z*. *jujuba*	Xinzheng, Henan	Xinzheng, Henan	N 34.47, E 113.88	G
47	Jixinzao	*Z*. *jujuba*	Xinzheng, Henan	Xinzheng, Henan	N 41.85, E 120.72	G
48	Bianhezao	*Z*. *jujuba*	Neihuang, Henan	Neihuang, Henan	N 35.94, E 114.82	D
49	Guangyangzao	*Z*. *jujuba*	JGSD	Zhengping, Henan	N 37.69, E 118.12	F
50	Dayewuhe	*Z*. *jujuba*	NKIJ (C129)	Neihuang, Henan	N 35.94, E 114.83	F
51	Tailihong	*Z*. *jujuba*	NKIJ (C250)	Henan	N 34.69, E 109.32	G
52	Henanlongzao	*Z*. *jujuba*	NKIJ (C375)	Henan/Beijing/Hebei	N 34.46, E 113.87	E
53	Ningyangliuyuexian	*Z*. *jujuba*	JGSD	Ningyang, Shandong	N 36.21, E 117.15	E
54*	Lingbaodazao	*Z*. *jujuba*	Lingbao, Henan	Lingbao, Henan	N 34.68, E 111.03	G
55	Lingbaosuanzao	*Z*. *acidojujuba*	Lingbao, Henan	Lingbao, Henan	N 34.65, E 110.93	G
56*	Jinancuisuanzao No.1	*Z*. *acidojujuba*	Jinan, Shandong	Jinan, Shandong	N 36.47, E 117.07	G
57	Cuisuanzao No.2	*Z*. *jujuba*	Jinan, Shandong	Jinan, Shandong	N 36.47, E 117.07	G
58	Jinansuanzao	*Z*. *acidojujuba*	Jinan, Shandong	Jinan, Shandong	N 36.53, E 117.23	G
59	Linyisuanzao	*Z*. *acidojujuba*	Linyi, Shandong	Linyi, Shandong	N 35.41, E 117.66	G
60*	Junzao	*Z*. *jujuba*	JESN	Jiaocheng, Shanxi	N 37.71, E 111.83	G
61	Linfentuanzao	*Z*. *jujuba*	JGSD	Linfen, Shanxi	N 35.89, E 111.79	G
62	Hamazao	*Z*. *jujuba*	JGSD	Yongji, Shanxi	N 34.91, E 110.38	G
63	Banzao	*Z*. *jujuba*	NKIJ (C296)	Jishan, Shanxi	N 35.63, E 110.9	D
64	Xiangzao	*Z*. *jujuba*	NKIJ (C118)	Yuncheng, Shanxi	N 35.14, E 110.94	E
65	Junzao No.1	*Z*. *jujuba*	Yan’an, Shaanxi	Shanxi	N 36.62, E 109.45	E
66	Junzao No.2	*Z*. *jujuba*	Yan’an, Shaanxi	Shanxi	N 36.62, E 109.45	G
67	Junzao No.3	*Z*. *jujuba*	Yan’an, Shaanxi	Shanxi	N 36.62, E 109.45	D
68	Dalishuizao	*Z*. *jujuba*	Dali, Shaanxi	Dali, Shaanxi	N 34.7, E 109.26	G
69	Bashenghu	*Z*. *jujuba*	Dali, Shaanxi	Dali, Shaanxi	N 34.7, E 109.84	G
70*	Yanchuangoutouzao	*Z*. *jujuba*	Yanchuan, Shaanxi	Yanchuan, Shaanxi	N 38.36, E 116.69	G
71	Jinzao (Heishan)	*Z*. *jujuba*	Changwu, Shaanxi	Changwu, Shaanxi	N 35.12, E 107.78	E
72*	Yanchuansuanzao No.1	*Z*. *acidojujuba*	Yanchuan, Shaanxi	Yanchuan, Shaanxi	N 36.8, E 109.96	D
73	Yichuanguoduxingsuanzao	*Z*. *acidojujuba*	Yichuan, Shaanxi	Yichuan, Shaanxi	N 36.14, E 110.25	G
74	Yanchuansuanzao No.2	*Z*. *acidojujuba*	Yanchuan, Shaanxi	Yanchuan, Shaanxi	N 36.8, E 109.96	G
75	Jiaxiangusuanzao	*Z*. *acidojujuba*	Jiaxian, Shaanxi	Jiaxian, Shaanxi	N 37.76, E 110.52	G
76	Huanglingsuanzao	*Z*. *acidojujuba*	Huangling, Shaanxi	Huangling, Shaanxi	N 35.54, E 109.44	D
77	Tuansuanzao	*Z*. *acidojujuba*	Jiaxian, Shaanxi	Jiaxian, Shaanxi	N 38.05, E 110.5	D
78*	Xiaohuipingguodusuanzao	*Z*. *acidojujuba*	Jiaxian, Shaanxi	Jiaxian, Shaanxi	N 38.07, E 110.51	G
79	Laoyasuanzoa	*Z*. *acidojujuba*	Qingjian, Shaanxi	Qingjian, Shaanxi	N 37.13, E 110.47	D
80	Qingjianxiaosuanzao	*Z*. *acidojujuba*	Qingjian, Shaanxi	Qingjian, Shaanxi	N 37.15, E 110.08	G
81	Mutiaozao	*Z*. *jujuba*	Yanchuan, Shaanxi	Yanchuan, Shaanxi	N 36.96, E 110.35	E
82	Huituanzao	*Z*. *jujuba*	Yanchuan, Shaanxi	Yanchuan, Shaanxi	N 36.88, E 110.31	G
83	Chunhuagusuanzao	*Z*. *acidojujuba*	Chunhua, Shaanxi	Chunhua, Shaanxi	N 34.85, E 108.42	D
84	Jinzao (Binxian)	*Z*. *jujuba*	Binxian, Shaanxi	Binxian, Shaanxi	N 35.08, E 108.01	E
85	Tongxinyuanzao	*Z*. *jujuba*	Tongxin, Ningxia	Tongxin, Ningxia	N 37.89, E 106.1	G
86	Zhongningxiaozao	*Z*. *jujuba*	Tongxin, Ningxia	Tongxin, Ningxia	N 37.11, E 105.79	G
87*	Lingwuchangzao	*Z*. *jujuba*	Lingwu, Ningxia	Lingwu, Ningxia	N 37.13, E 110.08	G
88	Heshuigusuanzao	*Z*. *acidojujuba*	Heshui, Gansu	Heshui, Gansu	N 35.8, E 108.08	B
89	Minqinxiaozao	*Z*. *jujuba*	NKIJ	Minqin, Gansu	N 38.67, E 103.29	G
90*	Xiaokouzao	*Z*. *jujuba*	Jingtai, Gansu	Jingtai, Gansu	N 37.05, E 104.33	G
91	Linzexiaozao	*Z*. *jujuba*	NKIJ (C131)	Linze, Gansu	N 39.28, E 100.23	G
92	Wenxianshatang	*Z*. *jujuba*	NKIJ (C216)	Wenxian, Gansu	N 32.97, E 104.73	G
93	Mingshandazao	*Z*. *jujuba*	NKIJ (C064)	Dunhuang, Gansu	N 40.24, E 94.64	G
94	Xinjiangxiaozao	*Z*. *jujuba*	NCGRI (ZF0229)	Shufu, Xinjiang	N 41.32, E 80.26	G
95	Hamidazao	*Z*. *jujuba*	NCGRI	Hami, Xinjiang	N 43.07, E 94.27	G
96*	Kuerlexiaozao	*Z*. *jujuba*	NCGRI (ZF0279)	Kuerle, Xinjiang	N 37.34, E 112.5	G

NCGRI: the National Crop (Jujube) Germplasm Resources Infrastructure(N 37.34, E 112.50), Taigu, Shanxi; NKIJ: National Key Base for Improved Chinese Jujube Cultivar, Cangzhou (N 38.36, E116.69), Hebei; JESN: Jujube experimental station of Northwest A&F University (N 37.13, E 110.09), Qingjian, Shaanxi; JGSD: Jujube Germplasm Repository of Shandong institute of pomology (N 36.21, E 117.15), Taian, Shandong.

These sample orders (11, 26, 34, 35, 36, 37, 54, 56, 60, 70, 72, 78, 87, 90, 96) tagged with * indicated those samples were firstly used for polymorphism detection. The coordinates of those samples from germplasm repository were given as one of the representative original site.

The collected leaves were stored in plastic bags filled with silicon balls and transported to the laboratory. Total genomic DNA was extracted from the silicon-dried leaves using a modification of a previously described cetyl-trimethylammonium bromide (CTAB) method [8] with a bead-beater (MM400; Restch, Haan, Germany).

### SSR locus search and primer design

The cpSSR loci were screened against an assembled draft chloroplast genome sequence of *Z*. *jujuba* cv. “Junzao” (File A in S1 Zip) using WebSat (http://wsmartins.net/websat) with the following repeat threshold settings: 10 repeats for mono-nucleotides, 6 for di-nucleotide SSRs, 5 for tri-nucleotide SSRs, 4 for tetra-nucleotide SSRs, and 3 for penta- and hexa-nucleotide SSRs. Polymerase chain reaction (PCR) primer pairs were designed from the sequences flanking the SSR loci using Primer3 [25]. Primer sets were found based on the following parameters: 20 to 24 base pairs (bp) in length, PCR product size of 100 to 300 bp in most cases, annealing temperature of 56°C to 60°C, and a GC content of 40% to 60%. One of the primers of each pair was located within a coding sequence to improve the PCR amplification success rate.

### Primer evaluation and detection of SSR polymorphisms

To evaluate the amplification efficiency of the primer sets for each locus, 15 cultivars/individuals (11 jujube and 4 wild jujube, as indicated in Table 1) from different regions were selected for PCR amplification. PCRs were performed in a 30-μL reaction mixture containing 1.5 μL of template DNA, 15 μL of 2×GoldStar Taq MasterMix (Kangwei, Beijing, China), 12.3 μL of deionized water, and 0.6 μL of forward and reverse primers (20μM). The PCR cycling conditions were as follows: 94°C for 5 min; 30 cycles at 94°C for 30 s, 57–59°C (melting temperature depends on the primer sets as listed in Table 2) for 30 s, and 72°C for 30 s; and a final extension at 72°C for 5 min. The PCR products obtained were verified by electrophoresis on 1.5% agarose gels under UV light. Fifteen SSR loci amplified efficiently and showed one solid band under UV light, and these products were further electrophoresed on 6.0% (w/v) denaturing polyacrylamide gels (PAGE) at 180 V for 2.5 h and visualized by silver staining to detect polymorphisms. For those markers showing polymorphisms, one representative PCR product of each allele was cut from the gel and purified using a QIAquick PCR Purification Kit (Qiagen, Limburg, Netherlands). The purified products were subcloned using a pUCm-T vector cloning kit (Sangon Biotech, Shanghai, China). Successful inserts (3 clones for each alleles) were sequenced using BigDye Terminator v3.1 Cycle Sequencing Kit (Applied Biosystems, Waltham, MA, USA) and electrophoresed on a 3130xl Genetic Analyzer (Applied Biosystems).

**Table 2:** Characteristics of 10 chloroplast microsatellite markers developed in *Ziziphus jujuba* and *Z*. *acidojujuba*.

SSR markers	Primer Sequence(5’→3’)	Repeats motif	Position	Expected size (bp)	Ta (°C)	*Na*	Allele size (bp)	*Ne*	*I*	*h*	*uh*
S1	F: GTCGTTTCGGGTTAAGAAGATG R: GAACTTGGTGGTTAAACTCTACT	(T)_17_	trnR-rrn5	270	57	3	268,270,275	1.065	0.159	0.061	0.062
S8	F: CCTTGATCCACTTGGCTACATC R: TCTAGCTGCGGTCGAAGTTC	(A)_12_	trnH-psbA	368	58	3	367,368,378	1.721	0.649	0.419	0.423
S12	F: ATCGGTTCAAATCCGATAAGG R: GATTCAATGGGTTAGGTCCACTT	(GTAGTAATTT)_1-2_ and (TCAATTAGA)_1-2_	trnT-psbD	394	58	3	371,394,404	1.088	0.197	0.081	0.081
S18	F: GTGATGGCTGGAGCAACAATAT R: CTCGTACATCTTTACCCTTGGC	(T)_10_	psaJ-rpl33	307	58	4	297,307,308,309	1.725	0.712	0.42	0.425
S24	F: TTGTTTCGCTCTTTATCTTCGG R: TTCGAATCCCTCTCTTTCCG	(A)_14_	psbI-trnS	216	59	3	214,215,216	1.31	0.433	0.237	0.239
S26	F: GAAACTCCAGAAAGGATGAAGA R: TCACGATTTCTAAAGTCGACGG	(TATATACGTATACGTACTGAAATACTAT)_1-3_ and (T)_10_	intro in trnL	356	59	4	328,354,380,382	1.769	0.747	0.435	0.439
S11	F: TCTACCGTTGAGTTAGCAACCC R: TTCTCCGTGCCATAGATTTGA	(A)_9_	trnK-rps16	222	58	2	222,223				
S14	F:CTCTACTGCGGTGACGATACTGT R:GTCGTTTCGGGTTAAGAAGATG	(T)_20_	rrn5-trnR	260	58	2	258,260				
S16	F: TTAGAGGGAGGGGTCAAACTTAT R: CGCATCTTCTCCTTGGCAA	3 indels: (TTATAT), (ATAATATT) and 200 bp deletion	psbZ-trnG	445	58	3	253,445,447				
YS-8	F: CGGTTTTCTACTAGCAGCTTTGAC R: GGTCCAATTGATCACCACGTC	(A)_9_C_2_ and (TCTGTGGTAGTTCATATATTTTA)_1-2_	petL-petG	384	58	2	361,384				

*Na*, observed number of allele; *Ne*, effective number of alleles; *I*, Shannonve number of alleles; *h*, diversity; *uh*, unbiased diversity. The alleles of S1, S8, S12, S18, S24, and S26 were detected in all 96 samples and one allele in for those markers was specific to *Ziziphus mauritiana*, while the alleles of S11, S14, S16 and Y-S8 were only detected in the 15 samples (11, 26, 34, 35, 36, 37, 54, 56, 60, 70, 72, 78, 87, 90, 96) of *Z*. *jujuba* and *Z*. *acidojujuba* as indicated in Table 1.

### Chloroplast diversity in *Z*. *jujuba* and *Z*. *acidojujuba*

To test the efficiency of the selected SSR markers, 96 samples (including the 15 previous samples) were further analyzed. The PCR reaction solution was also prepared for each marker independently as the above with the exception that the forward primer was labeled with florescent dyes (FAM (S1, S18), HEX (S8, S24), TAM (S12, S26)). Then, a touch-down PCR procedure was followed for each marker, i.e., 94°C for 5 min, 10 cycles from 60°C to 55°C (94°C for 30 s, 60°C for 30 s, 72°C for 30 s), followed by 20 cycles (94°C for 30 sec, 55°C for 30 s, 72°C for 30 s), and a final extension of 6 min at 72°C.The PCR products were electrophoresed on an ABI 3130xL genetic analyzer (Applied Biosystems) using the “fragment analysis” function. The amplified results were collected and analyzed with Genemapper (Applied Biosystems).

### Data analysis

The number of observed alleles (*Na*) for each SSR locus was counted for all *Ziziphus* samples. The effective number of alleles (*Ne* = 1 / (Σ*P*_*i*_^*2*^), the Shannon index (*I*) = –ΣP_i_ ln *P*_*i*_, *P*_*i*_: the frequency of the *i* allele), diversity index (*h* = 1 – ΣP_i_^2^), and unbiased diversity (*uh* = (N / (N– 1) *h*) were calculated using GenAlEx 6.5 [25]. A cluster analysis on different samples was performed by calculating the Jaccard coefficient based on the 0/1 matrix data (96 samples vs. 20 alleles) using PAST 3.04 [26]. To demonstrate the evolutionary pattern of jujube, the relationships between the different chlorotypes of jujube and wild jujube were analyzed based on the difference in the repeat number of mononucleotide residues (A and/or T) in cpSSR loci using a median network joining method with NETWORK 4.6.1.3 [27].

## Results

### cpSSR polymorphism detection

**Fig 1:**
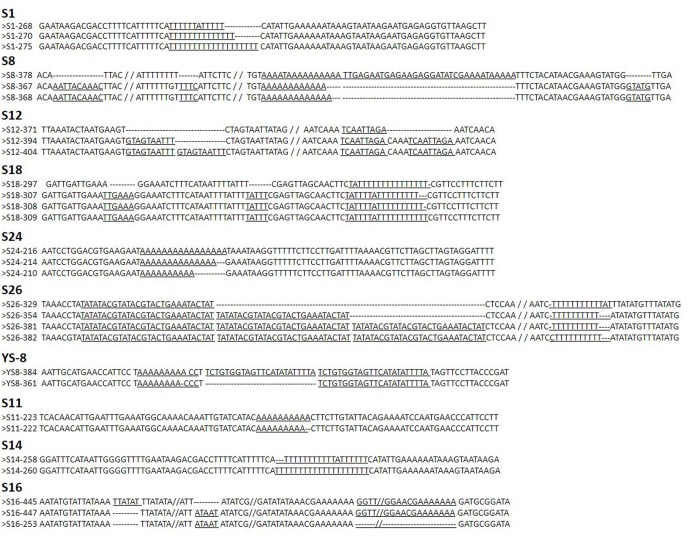
Sequence variation of the alleles detected in 10 cpSSRs.

Ninety-six SSRs were identified in the jujube chloroplast genome, 90 of which were composed of A or T. Seventy-three SSRs occurred in intergenic spacer regions (IGS), 19 in introgenic spacer regions, and 4 in coding sequence regions (1 in rpoB and 3 in ycf1). A total of 46 SSR loci located in IGS regions were selected for the detection of polymorphisms (S1 Table). Of the 46 SSR loci, 38 were amplified well using the designed primer pairs (S1 Table). Next, 32 primer sets were selected to detect polymorphisms by the silver staining of 15 samples. The PAGE results revealed 10 loci (S1, S8, S11, S12, S14, S16, S18, S24, S26, and Y-S8) that contained more than one allele. Sequencing of those alleles, as seen in Fig 1 (full sequences in File B in S1 Zip), revealed seven loci (S1, S8, S11, S14, S18, S24, and S26) with typical mononucleotide tandem repeats (A/T), however, S12, S16, and YS-8 were attributed to indels or variation of long repeat motif. Particularly, S26 sequence contained two polymorphic loci, one of which was a repeat motif of 26 bp (TATATACGTATACGTACTGAAATACTAT), and mononucleotide tandem repeats; these were treated as two separate loci (one long motif repeats and one A/T repeat motif) in later analyses. In addition, 2 or 4 special indels were present in S8 (4 deletion), S12 (2), and S18 (2) loci for *Z*. *mauritiana*. Finally, six loci were selected for further analysis (S1, S8, S12, S18, S24, and S26) on 96 samples using ABI genetic analyzer.

### Characterization of cpSSR markers

In total, 20 alleles were detected in 6 cpSSR loci among the 96 samples. As listed in Table 2, the *Na* ranged from 3 to 4, and the *Ne* was calculated as 1.065 to 1.769. *Ziziphus mauritiana* always contained a specific allele at each locus from two other species. Thus, two to three alleles per locus were observed at six loci among jujube and wild jujube, but not in Indian jujube. In general, the *I* for each cpSSR ranged from 0.159 to 0.1747, and the diversity indices (*h*) and *uh* were 0.061 to 0.435 and 0.062 to 0.439, respectively. The diversity indices for jujube and wild jujube were calculated as *Ne* = 1.506, *I* = 0.460, and *h* = 0.280. These indices were also calculated for wild jujube and jujube, and the results showed higher values for wild jujube (*Ne* = 1.619, *I* = 0.483, and *h* = 0.303; *n* = 23) than jujube (*Ne* = 1.393, *I* = 0.437, and *h* = 0.256; *n* = 72) (Table 3).

**Table 3:** Genetic diversity indices of the populations of wild jujube and jujube.

Population	*N*	*Na*	*Ne*	*I*	*h*	*uh*
jujube	72	2.833	1.393	0.437	0.256	0.260
Sour jujube	23	2.000	1.619	0.483	0.303	0.317
Total	95	2.417	1.506	0.460	0.280	0.288

### Chlorotype and genetic relationships among the jujube and wild jujube

**Fig 2:**
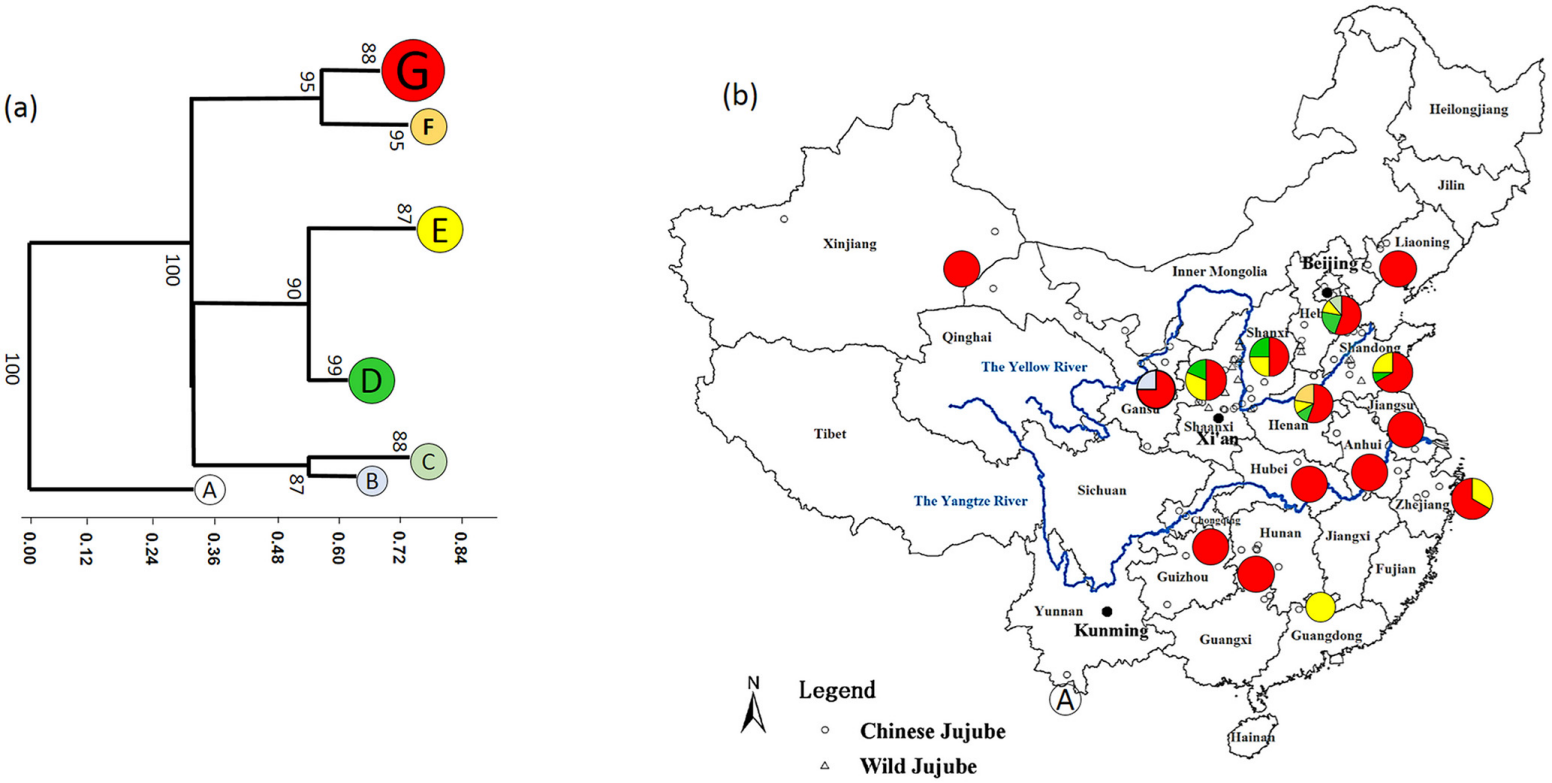
A, Cluster analysis of the 7 chlorotypes detected in the 96 samples based on Jaccard Coefficient (bootstrap = 1000). Chlorotype A includes 1 cultivar of Ziziphus mauritiana. One wild jujube individual in B, 2 wild jujube individuals in C, 5 jujube cultivars and 7 wild jujube individuals in D, 12 jujube cultivars in E, 2 jujube in F; 53 jujube and 13 wild jujube in G. The cultivar/individual information included in each chlorotype were listed in Table 1. B, Original locations of samples and geographic distributions of the chloroplast haplotypes found in each province. The pie charts on map represent the chlorotype composition of the samples from the corresponding province (Hebei, Tianjin and Beijing were pooled, eastern Gansu and Ningxia were pooled) and the color in each chart represent the chlorotype as indicated in the cluster tree.

The cluster analysis on 96 samples based on the 20 alleles in 6 cpSSR loci revealed 7 chlorotypes in all 96 samples (Fig 2A). Indian jujube with one sample had a distinct chlorotype A. Both jujube and wild jujube had two specific chlorotypes, i.e., E (12 cultivars) and F (2 cultivars) for jujube, and B (1 individual) and C (2 individuals) for wild jujube, respectively, and they also shared other two chlorotypes (D and G). Chlorotype G was the dominant type in jujube and wild jujube, it accounted for 53 of 72 jujube cultivars and 13 of 23 wild jujube individuals. Chlorotype D contained 7 individuals of wild jujube and 5 cultivars of jujube. The jujube and wild jujube were clustered into 3 groups, i.e., I (G and F), II (E and D), III (B and C) at coefficient < 0.5 supported by the high values of bootstrap (Fig 2). The two wild-jujube-specific chlorotypes (B and C) grouped into one dependent cluster III, while other two groups contained one jujube-species chlorotype and a jujube/wild jujube mix chlorotype.

**Fig 3:**
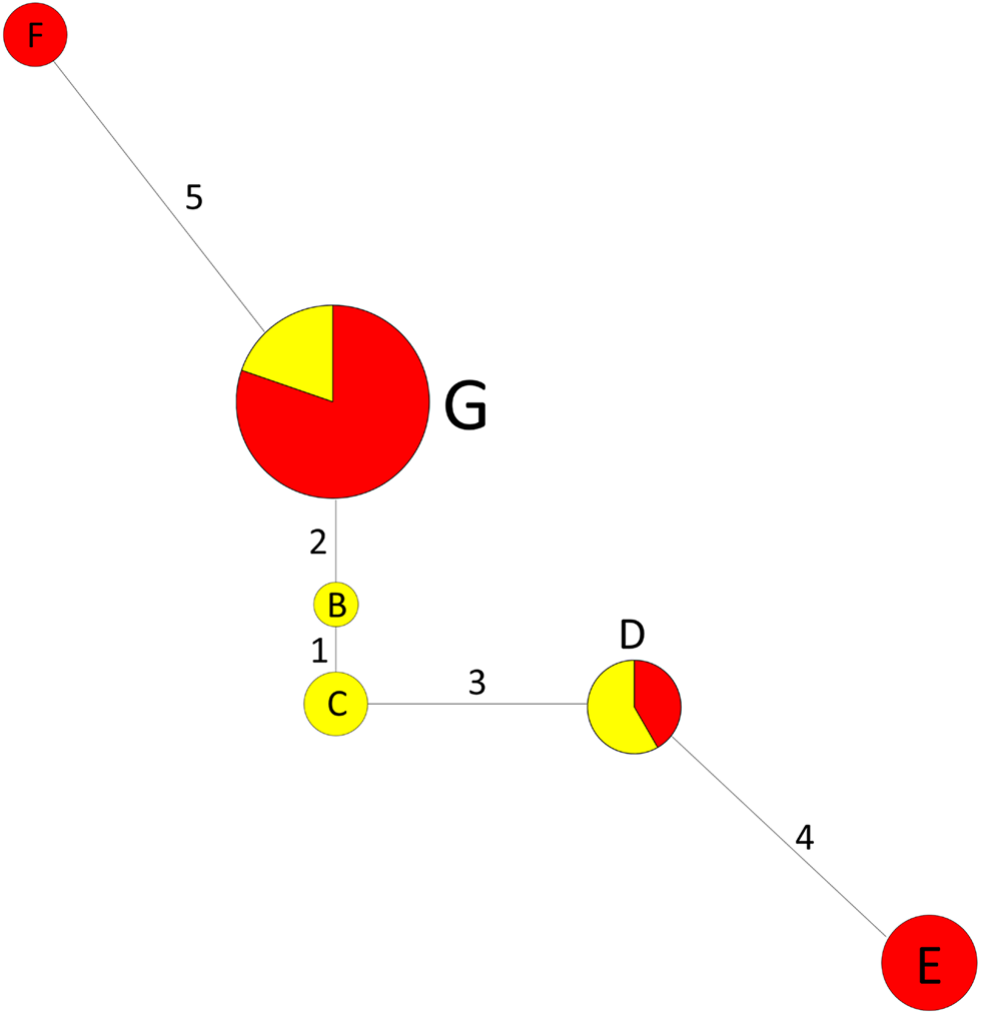
Chlorotype median network showing six chlorotypes identified in *Ziziphus jujuba* and *Z*. *acidojujuba* based on stepwise differences of five cpSSR loci. The circle size indicates the chlorotype frequency in 95 samples of Z. jujuba and Z. acidojujuba, the red color in the pie chart shows the proportion of jujube, and the yellow color shows the proportion of wild jujube. Number on the branch nodes represent the mutation steps based on the difference in the repeat number of cpSSR loci. One wild jujube individual in B, 2 wild jujube individuals in C, 5 jujube cultivars and 7 wild jujube individuals in D, 12 jujube cultivars in E, 2 jujube in F; 53 jujube and 13 wild jujube in G.

Chlorotype network analyses were performed based on the five cpSSR loci (S1, S8, S18, S24, and S26) comprising typical mononucleotide tandem repeats in jujube and wild jujube. As seen in Fig 3, two wild-jujube-specific chlorotypes (B and C) with only one mutation step were located centrally in the network. The network extended in two directions; i.e., B (wild jujube) **→** G (wild jujube/jujube) **→** F (jujube), and C (wild jujube) **→** D (wild jujube/jujube) **→** E (jujube).

### Distribution of chlorotypes

As showed in Fig 2B, the most frequent chlorotype, G, contained 65 samples (13 wild jujube and 53 jujube) that were present throughout all provinces where jujube was traditionally cultivated. In addition, the jujube cultivars planted in the southern regions of the Yangtze River (Yunnan, Guangdong, Guangxi, Fujian, Jiangsu, Hunan, Zhejiang, Anhui, Chongqing, and Hubei) as well as northeast (Liaoning) and northwest (Xinjiang, Ningxia, and western Gansu) far from the Yellow River also had the common G chlorotype, except for two cultivars with chlorotype E from Zhejiang and Guangdong Province. The next most frequent chlorotypes, D and E occurred from northern Shaanxi to Shandong Province along the Yellow River. The two rare chlorotypes associated with wild jujube, chlorotypes B and C, were distributed in Hebei Province and eastern Gansu Province, respectively, showing long-distance isolation. The rarest chlorotype of jujube, F, occurred in two cultivars in Henan Province.

## Discussion

In this study, 46 primer sets flanking the SSR loci were designed; 38 amplified well, demonstrating the efficiency of the PCR amplification method. This confirmed that *de novo* sequencing of the chloroplast genome is an ideal method for the development of SSR markers [21]. Six cpSSR markers showed a total of 14 alleles in jujube and wild jujube, which suggested a high level of sequence conservation between closely related species; i.e., jujube and wild jujube. Notably, the polymorphic SSR markers could also be amplified in *Z*. *mauritiana*, indicating that they could be used as cross-species markers within *Ziziphus*. However, higher differentiation was observed between Indian jujube and jujube/wild jujube due to the presence of indels and mutation, suggesting a more distant relationship.

The traditional geographical distribution range of jujube covered that of wild jujube, but both of them highly cauterized in the area from the mid- (north Shaanxi) to lower reaches (Shandong Province) of the Yellow River in China. Correspondingly, all six chlorotypes occurred frequently in those regions. Those jujube cultivars grown more distant from the Yellow River did not reveal distinct chlorotypes; instead, almost all belonged to chlorotype G. Accordingly, we might consider that jujube originated from the mid-to lower reaches of the Yellow River based on the distribution pattern of jujube and wild jujube chlorotype. The cultivars collected from southern, northeastern, and northwestern China were likely introduced from the distant reaches of the Yellow River, coinciding with historical Chinese migrations [28]. In addition, the mid-reaches of the Yellow River canyon, located between Shanxi and Shaanxi Provinces, were considered to be the sites of primary origin because of the diverse germplasm [3]. However, these regions (north Shaanxi, Shanxi, and eastern Gansu) did not have higher chlorotype diversity than the lower reaches (Henan, Hebei, and Shandong Provinces). This might imply that jujube originated from a more extended area; e.g., the mid- and lower reaches of the Yellow River.

In this study, jujube cultivars and wild jujube individuals were not completely classified into two groups based on the cluster analysis (Fig 2A). As showed in Fig 2A, six chlorotypes belonging to jujube/wild jujube can be clustered into three groups, Group I (chlorotype F and G) and Group II (D and E) include jujube and wild jujube, while Group III (B and C) was specific to wild jujube (Fig 2A). A previous study on the relationships between 25 jujube cultivars and 19 wild jujube individuals from north Shaanxi used RAPD markers to cluster the samples into 3 groups, each including both wild jujube and jujube, rather than one group of wild jujube and another of jujube [29]. Although other studies investigating the relationships between different jujubes also included some wild jujube individuals, the wild jujubes were placed into different groups of jujube cultivars [6]. In 2009, Li et al. [30] analyzed 14 species of *Ziziphus* originating from China using SRAP markers and showed that jujube and wild jujube should be treated equally as two subspecies of *Z*. *jujuba* (*Z*. *jujuba* Mill. subsp. *jujuba* for jujube and *Z*. *jujuba* Mill. subsp. *spinosa*) due to their close genetic relationship. This demonstrated a close relationship between jujube and wild jujube, which could not be clearly separated.

On the other hand, it was long considered that jujube was domesticated from wild jujube in several directions [3]. According to the network analysis in this study (Fig 3), two directions from two wild-jujube-specific chlorotypes (B and C) to jujube chlorotypes (E and F) indicated that there might be at least two way for jujube domestication from wild jujube. The two wild-jujube-chlorotypes (B and C) centered in the network were located in Hebei (lower-reach of Yellow River) and eastern Gansu Provinces (Mid-reach), respectively. This result was also similar with the analyses of wild jujube populations based on nuclear SSR markers conducted in a previous study [8] to some extent, in which two diversity centers were revealed in mid-reach and lower-reach of Yellow River. Thus, independent origins and domestication way could be presumed for jujube domestication. Undoubtedly, these should be confirmed by analyses of intense samples of jujube and wild jujube collected from the distribution region in future research.

In conclusion, 10 polymorphic cpSSR markers were developed based on the jujube chloroplast genome, 7 of which contained typical mononucleotide tandem repeats. In total, 7 chlorotypes were identified in the 96 samples based on 6 markers. Indian jujube was distinct from the other samples. Jujube and wild jujube contained 4 chlorotypes and shared 2 chlorotypes, primarily chlorotype G, which was also widely distributed on a national level (53 of 72 jujube cultivars and 13 of 23 wild jujube individuals). Network analysis revealed there are at least two domestication way of jujube from wild jujube. A more extensive study including wild jujube and jujube samples from all of the distribution regions is needed to find cogent evidence supporting the origin and evolution of jujube, which is closely connected with traditional Chinese culture.

## Supporting Information

S1 Table PCR amplification and polymorphism of the intergenic region. (XLSX)

S1 Zip Contains File A, “Chloroplast draft genome sequence of *Ziziphus jujuba* cv. “Junzao”, and File B, “Sequences of detected alleles in 10 cpSSRs”. (ZIP)
